# Monitoring training activity during gait-related balance exercise in individuals with Parkinson’s disease: a proof-of-concept-study

**DOI:** 10.1186/s12883-017-0804-7

**Published:** 2017-01-31

**Authors:** David Conradsson, Håkan Nero, Niklas Löfgren, Maria Hagströmer, Erika Franzén

**Affiliations:** 1 Department of Neurobiology, Care Sciences and Society, Division of Physiotherapy, Karolinska Institutet, Stockholm, Sweden; 2 Functional Area Occupational Therapy & Physiotherapy, Allied Health Professionals Function, Karolinska University Hospital, Stockholm, Sweden

**Keywords:** Accelerometry, Balance exercise, Dual-task, Postural control, Training progression, Wearable sensors

## Abstract

**Background:**

Despite the benefits of balance exercise in clinical populations, balance training programs tend to be poorly described, which in turn makes it difficult to evaluate important training components and compare between programs. However, the use of wearable sensors may have the potential to monitor certain elements of balance training. Therefore, this study aimed to investigate the feasibility of using wearable sensors to provide objective indicators of the levels and progression of training activity during gait-related balance exercise in individuals with Parkinson’s disease.

**Methods:**

Ten individuals with Parkinson’s disease participated in 10 weeks of group training (three sessions/week) addressing highly-challenging balance exercises. The training program was designed to be progressive by gradually increasing the amount of gait-related balance exercise exercises (e.g. walking) and time spent dual-tasking throughout the intervention period. Accelerometers (Actigraph GT3X+) were used to measure volume (number of steps/session) and intensity (time spent walking >1.0 m/s) of dynamic training activity. Training activity was also expressed in relation to the participants’ total daily volume of physical activity prior to the training period (i.e. number of steps during training/the number of steps per day). Feasibility encompassed the adequacy of data sampling, the output of accelerometer data and the participants’ perception of the level of difficulty of training.

**Results:**

Training activity data were successfully obtained in 98% of the training sessions (*n* = 256) and data sampling did not interfere with training. Reflecting the progressive features of this intervention, training activity increased throughout the program, and corresponded to a high level of the participants’ daily activity (28–43%). In line with the accelerometer data, a majority of the participants (*n* = 8) perceived the training as challenging.

**Conclusions:**

The findings of this proof-of-concept study support the feasibility of applying wearable sensors in clinical settings to gain objective informative measures of gait-related balance exercise in individuals with Parkinson’s disease. Still, this activity monitoring approach needs to be further validated in other populations and programs including gait-related balance exercises.

**Trial registration:**

NCT01417598, 15th August 2011.

## Background

Training interventions are often based on frameworks, including theoretical principles, that underpin and guide the practical execution of training [1]. Although the transferability of these principles into practice is important for internal validity, the characteristics of training are often poorly described [2]. In the light of uncertainty that exists regarding the content of training, stratifying different training programs or establishing dose-response relationships for balance training may not be possible [2].

Incomplete descriptions of training programs are particularly problematic in balance training, which in contrast to, for example, strength and aerobic training, [3] lacks a standardised approach regarding the monitoring of training [4–6]. Instead, the content of balance training is often described with subjective and generic descriptions (e.g. duration of the training period, number of training sessions and the type of exercise performed); [7, 8] thus, providing limited information on the actual training stimuli. This uncertainty is further aggravated by the fact that several different impairments are targeted through balance training. For example, stationary exercises (e.g. controlling centre of mass during weight shift exercises), walking exercises (e.g. obstacle courses) along with cognitive-motor interference, e.g. dual-tasking (DT, the concurrent performance of two different tasks), are common targets in balance training [9–11]. Furthermore, although the principle of progressive overload (i.e. exercises need to reflect the limits of individual capacity with a gradual increase of training load) is a well-established concept [3], the conceptualisation of this principle in balance training is unclear [4, 5]. For instance, in a recent meta-analysis investigating dose-response relationship of balance training, the poor descriptions of training across studies did not afford the authors to conclude upon the optimal training volume and intensity [8]. Thus, as information on training stimuli is a prerequisite for adequate evaluations of dose-response relationship, investigating alternative methods that could provide detailed indicators of balance training was recommended [7].

The focus of this study was to explore the potential benefits of using an objective approach to specifically monitor the dynamic component (i.e. gait-related exercises) of balance training. Wearable sensors (e.g. accelerometers), often used to measure movements associated with physical activity in daily living, provide an objective and direct measurement of the activity, such as volume (e.g. number of steps per day) and the time spent at different intensity levels [12, 13]. Since walking exercises is an important element of balance training [14, 15], wearable sensors could potentially be used to complement subjective evaluations, i.e. by providing informative measures of the dynamic aspect of balance training (e.g. volume and intensity). The rationale for this approach is supported by the close link between balance control and dynamic activity. Indeed, wearable sensors have previously been used to provide real-time feedback of balance training [16, 17]. However, to our knowledge, no previous studies have used wearable sensors for the monitoring of gait-related balance exercise.

For individuals with Parkinson’s disease (PD), impaired balance control leads to activity limitations and fall-related injuries [18–20]; and to combat these problems, challenging balance training is recommended [21, 22]. Specifically, DT has shown to degrade balance and gait performance in individuals with PD, resulting in vulnerability to falls during many daily activities [23–25]. Furthermore, DT training has shown positive effects on gait and balance performance in individuals with PD [26–28] thus, recommended to be implemented in clinical practice [29]. With this in mind, our group have developed a specific training program for individuals with PD aiming to target highly challenging and progressive training conditions, which incorporated DT-exercises [30]. In previous work, we found that the participants perceived this training program to be progressively challenging [31] with positive effects on balance, gait and physical activity [28]. As a first step towards establishing objective indicators for gait-related balance exercise, we aimed to investigate the feasibility of wearable sensors in monitoring key elements (i.e. levels and progression of training activity) of this program in individuals with PD.

## Methods

### Study design

This study, approved by the Regional Board of Ethics in Stockholm, Sweden (approval no: 2006/151-31, 2010/1472-32, 2012-1829-32), was carried out as a longitudinal pretest-posttest design. The present study is a sub-project of a randomised controlled trial that studied the effects of balance training in individuals with PD. The main study has been described in a previous publication [30] and was registered at https://clinicaltrials.gov/ (BETA-PD study, NCT01417598).

### Participants

Ten individuals with mild to moderate PD were recruited via advertisements in local newspapers, from Karolinska University Hospital and outpatient neurological clinics in Stockholm County. The study sample was a convenience sample from the randomised controlled trial [30]. Inclusion criteria were: a clinical diagnosis of idiopathic PD according to the Queens Square Brain Bank criteria; [32] Hoehn and Yahr stage two or three; [33] ≥60 years of age; the ability to independently ambulate indoors without a walking aid, and ≥ 3 weeks of stable anti-Parkinsonian medication prior to enrolment. Exclusion criteria were: a Mini Mental State Examination score of <24, [34] or a medical condition (other than PD) that influenced balance performance. Eight of the participants were taking carbidopa/levodopa medications, seven were taking dopamine agonists and four were taking monoamine oxidase type B or catechol-O-methyl transferase inhibitors. All participants provided written informed consent prior to entering the study.

### Balance training

The participants were enrolled in two training groups (group size: six and four) that participated in a 10-week training intervention (1 h, three times per week) [30]. All participants followed their normal scheme for PD medication and the balance training was performed in the ON-medication state.

Every training session began with a five minute warm-up, consisting of varied walking tasks aimed at boosting the cardiovascular system. The following 50 min, including short resting periods, focused on highly-challenging exercise blocks (approximately 10 min per block) of standing and walking conditions. Specifically, four balance components were addressed in this program: (1) *Sensory integration* (walking tasks on varying surfaces with or without visual constraints), (2) *Anticipatory postural adjustments* (voluntary arm/leg/trunk movements, postural transitions, and multidirectional stepping, emphasising movement velocity and amplitude), (3) *Motor agility* (inter-limb coordination under varying gait conditions and quick shifts of movement characteristic during predictable and unpredictable conditions), and (4) *Stability limits* (controlled leaning tasks performed while standing with varying bases of support, stimulating weight shifts in multiple directions). The program ended with a 5-min cool-down session of slow walking, axial stretching and breathing exercises. The exercise principles and objectives for the balance components have been detailed in the study protocol [30].

To promote training progression and motor learning, the intervention period was divided into three blocks (A, B and C) [31]. *Block A* (weeks 1–2): participants were introduced to the single-task exercises of each balance component separately, with an emphasis on movement quality, the objectives of the exercises, as well as task-specific motor learning. *Block B* (weeks 3–6): basic DT-exercises were introduced (i.e. cognitive or motor secondary task) and comprised approximately 40% of each session [28]. While addressing each balance component separately during this block, the level of difficulty and task variation was increased. *Block C* (weeks 7–10): the level of difficulty of all exercises was further enhanced by increasing the variation by combining several balance components during exercise. Additionally for DT-exercise, motor and cognitive secondary tasks were combined during the same exercise session, and the time spent on DT-exercises was increased (approximately 60% of each session) [28]. Furthermore, the amount of dynamic balance exercise (e.g. walking exercises, obstacle course) in relation to stationary exercises was gradually increased across the three blocks based on the capacity of the training group.

The balance training was supervised by two physiotherapists (all with similar experience in rehabilitation and managing individuals with PD) in a university hospital setting. To achieve an adequate level of challenge, single task exercises were adapted to a level where the participants had to rely on the use of reactive postural adjustments (e.g. side stepping) to control their balance during voluntary movements. Importantly, no external perturbations were given by the trainers; instead reactive postural adjustments occurred due to highly challenging training conditions. The adjustments of the level of difficulty were performed by the trainers and the level of difficulty was increased if postural reactions were absent and decreased if exercises caused excessive postural instability. Similarly, the level of difficulty for DT exercises aimed to inflict an interference of motor performance (e.g. decreased walking velocity). Importantly, the modifications of training relied upon the physiotherapists’ clinical judgment, and were continuously re-evaluated based on the participant’s performance throughout the program. Accordingly, in achieving consistent application of theoretical principles, [30] the physiotherapists took part in two 4-h education sessions of both theory and practice.

### Data collection and management

Training activity was measured with accelerometers (Actigraph GT3X+, Pensacola, FL, USA). The GT3X+ is a lightweight (19 g) accelerometer that records acceleration in three axes (vertical, antero/posterior, and medio/lateral) with a sampling frequency of 30 Hz. The GT3X+ has been found valid for the assessment of physical activity in healthy adults [35, 36] with an inter-instrument correlation coefficient between 0.90 – 0.99 [37] and an intra-instrument coefficient of variation of ≤2.5% [38]. The output is converted to an arbitrary unit (counts) in either axis or as a composite vector magnitude (i.e. the resultant of the magnitude of all three axes). The participants were equipped with accelerometers during all training sessions (i.e. 30 sessions with continuous data acquisition for 60 min). The sensors were placed around the hip slightly above the iliac crest over the lateral side of the left hip, to catch large body movements and minimise the influence of tremor. The physiotherapists were responsible for managing the accelerometers during training, and the data were downloaded by one of the study coordinators once per week. Prior to the intervention period, accelerometers were also used to measure the physical activity level (i.e. data acquisition during all wake time of the day) under free-living conditions [39]. The outcome, average steps per day, was based on 4 – 7 days of at least 9 h of valid data [13]. The requirement for 4 recording days was based on previous recommendations for healthy adults [13] and research on individuals with PD [39].

Data were processed and summarised in 15-s epochs by using the ActiLife six software. The ActiLife default filter setting was used, according to previously published recommendations [40]. As proxies for training activity, the number of steps per session, the time spent walking slowly (<1.0 m/s) and brisk walking (>1.0 m/s) were used to reflect the volume and intensity of dynamic exercises, respectively. The number of steps per session was derived from the vertical axis (using a default logarithm in the ActiLife software), and the time spent walking slowly or in a brisk pace were based on the vector magnitude. Vector magnitude cut-points have been developed for individuals with mild to moderate PD, i.e. 50 – 470 vector magnitude counts per 15 s indicated walking <1.0 m/s and ≥470 vector magnitude counts per 15 s indicated walking >1.0 m/s [41]. These cut-points have been established using Receiver Operating Characteristic Curve analysis to identify different walking speeds in individuals with PD [41]. To exclude sedentary time from slow walking (<1 m/s) we used established cut-points (i.e. 50 counts per 15 s) [42].

### Feasibility of wearable sensors to monitor balance training activity

Feasibility encompasses the adequacy of data sampling and data output. The adequacy of data sampling was indicated by the proportion of training sessions with valid data and the physiotherapists’ perception of using the accelerometers as well as the potential interference effect of this evaluation on training. Regarding the adequacy of data output, we focused on the levels and progression of training activity as they both are key elements of this training program [30, 31]. Outcomes for the levels of training were the average volume (steps per session) and intensity (time spent walking <1.0 m/s and >1.0 m/s) of training activity across the training program (i.e. Block A, B and C) were used. To gain further insight into the levels of training activity, training volume was also expressed as the percentage of the participant’s total daily volume of physical activity (i.e. steps during training/the number of steps per day). Training progression was evaluated by comparing the levels of activity between the blocks (i.e. A vs. B and B vs. C), with Wilcoxon signed-rank tests (level of significance: *p* ≤ 0.05) used as test statistic. To assess the participants’ perception of the level of difficulty of the training, the participants’ responded to a question (“*How do you perceive the level of challenge of the balance training?”*) on a four point scale (1) “too easy”, 2) “easy”, 3) “challenging”, and 4) “very challenging”) [31]. If responding three or four, the participants were categorized as perceiving the training as challenging.

## Results

Participant characteristics, severity of motor symptoms, clinical characteristics, history of falls, fear of falling, gait speed and physical activity level prior to the intervention are presented in Table 1. Throughout the training period, two adverse events (both were falls during training) were reported. None of these events caused injury or pain that interfered with the participants’ ability to proceed with the balance training or other activities.

**Table 1:** Participant characteristics

					UPDRS motor part III^a^										
Id	Sex	Age (yrs)	BMI	Duration of PD (yrs)	Tremor	Rigidity	Bradykinesia	Total	H&Y	Previous falls^b^	No of falls	FOG^c^	Fear of falling^d^	Gait speed (m/s)^e^	Physical activity (steps per day)^f^
1	F	66	17.4	5	4	7	20	41	2	Y	1	N	N	1.27	3656
2	M	72	23.5	1	2	13	21	32	2	N	–	N	N	0.80	2157
3	M	80	22.8	5	2	18	16	33	3	Y	12	N	Y	1.29	1343
4	M	65	27.1	1	3	6	21	50	2	N	–	N	N	1.53	8728
5	F	72	22.9	2	3	5	16	45	3	Y	1	N	Y	1.22	6593
6	F	83	23.5	2	9	7	14	48	3	N	–	N	Y	0.97	781
7	M	74	26.0	2	10	6	20	38	2	Y	6	N	N	1.16	1553
8	M	69	25.4	5	7	8	11	31	2	Y	3	Y	N	1.13	3258
9	M	63	21.9	6	8	15	20	47	2	Y	2	N	N	1.40	7558
10	M	70	26.0	7	8	12	19	39	2	N	–	N	Y	1.13	10696
Mean	–	71	23.8	3	6	10	18	40	–	–	–	–	–	1.19	4632

*Abbreviations*: *BMI* Body Mass Index; *PD* Parkinson’s disease; *UPDRS* Unified Parkinson’s Disease Rating Scale; *H&Y* Hoehn and Yahr, *FOG* freezing of gait

^a^Maximum total score is 108 points (higher score = higher severity of motor symptoms). Sub-scores were calculated as follows: tremor; sum of item 20–21, rigidity; sum of item 22, and bradykinesia; sum of item 23–26 and item 31

^b^Falls assessed retrospectively the previous 12 months and defined as an unexpected event in which the participants came to rest on the ground, floor, or lower level

^c^Freezing of gait assessed by item 14 of UPDRS activities of daily living part II (a score ≥2 indicated freezing of gait)

^d^Fear of falling assessed with a single-item question (“*In general, are you afraid of falling?*”)

^e^Self-selected gait speed assessed with an electronic walkway system (GAITRite® system, CIR Systems, Inc., Haverton, PA, USA)

^f^Physical activity level assessed by accelerometry (Actigraph GT3X+) in free-living conditions during 4 – 7 consecutive days

### Adequacy of data sampling

The average training attendance rate was 87% (range: 63 – 100%). In all, participants took part in a total of 261 training sessions, and valid training activity data were obtained from 256 sessions (98%). Missing data (*n* = 5) were only reported when a participant left before the training session ended. The objective measurement of training activity did not interfere with the supervision of training or the participant’s performance of balance exercises.

### Adequacy of data output

**Fig. 1:**
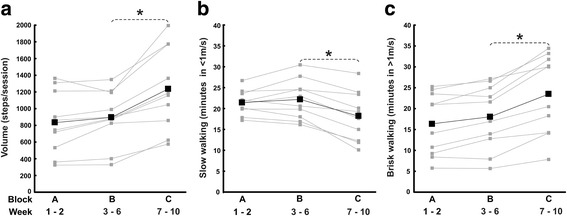
The level of dynamic exercises throughout the 10-week intervention. **a** Volume (steps/session), **b** Slow walking (minutes in <1 m/s) and **c** Brisk walking (minutes in >1 m/s) plotted as group and individual mean values for Block A (week 1–2), Block B (week 3–6) and Block C (week 7–10). ^*^ *P* ≤ 0.05

**Fig. 2:**
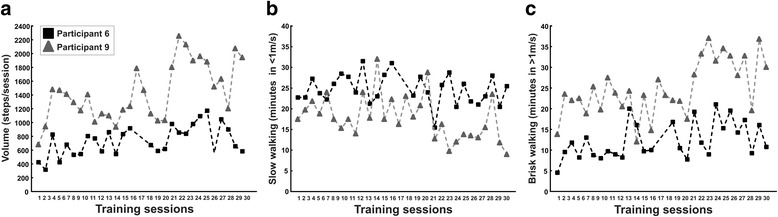
Individual data of training activity. Typical patterns of **a** Training volume, **b** Slow walking (minutes in <1 m/s) and **c** Brisk walking (minutes in >1 m/s) plotted for all 30 trainings sessions for a low performance (participant six) and high performance individual (participant nine)

The average (min-max) steps per training session were 833 (323–1365) in Block A, 895 (329–1348) in Block B and 1237 (576–2000) in Block C (see Fig. 1a). The number of steps performed during Block A, B and C corresponded in average (min-max) to 28% (8–68%), 33% (10–106%) and 43% (13–110%) of the participants’ total daily activity, respectively. For the 60 min duration of each training session, the average minutes spent walking slowly (<1.0 m/s) were 21 (35%) in Block A, 22 (37%) in Block B and 18 (30%) in Block C and the average minutes spent walking in a brisk pace (>1.0 m/s) were 16 (27%) in Block A, 18 (30%) in Block B and 23 (38%) in Block C (see Fig. 1b, c). The number of steps, the time spent in slow walking (<1.0 m/s) and brisk walking (>1.0 m/s) – representing training progression – remained unchanged between Block A and B (*p* > 0.333), whereas a significant increase occurred for the number of steps and time spent in brisk walking between Block B and C (*p* = 0.005). In contrast, the time spent walking slowly decreased significantly between Block B and C (*p* = 0.005). In line with the objective data of training activity, during most of the intervention period, the majority of participants (*n* = 8) rated the training as challenging. Typical patterns of training volume and the time spent in slow and brisk walking for a low performance (participant six) and high performance individual (participant nine) are presented in Fig. 2a, b, c.

## Discussion

This study aimed to investigate the feasibility of using wearable sensors to provide objective indicators of the levels and progression of training activity during gait-related balance exercise in individuals with Parkinson’s disease. The results demonstrated that: 1) it was feasible to use wearable sensors to collect data of training activity, and 2) the sampled data reflected the progressive feature of this intervention. The findings of this study support the feasibility of applying wearable sensors in clinical settings to gain objective informative measures of gait-related balance exercise.

We believe that objective measurements of gait-related balance exercise could improve the description of training and potentially also help establishing future dose-response for training program emphasizing walking abilities. Specifically, data on training activity were successfully sampled with the accelerometers, and the physiotherapists and participants, respectively, found it simple to administer and wear. For instance, after being introduced to the sensors by the physiotherapists, participants quickly adapted to them, which became a natural and integrated part of the training procedure. The clinical utility of wearable sensors as a source for feedback for promoting physical activity has previously been reported in individuals with PD [43]. In line with the framework of this training program [30, 31], and objective data on training activity demonstrated a progressive pattern of activity throughout the program (Fig. 1a, b), which corresponded to a high level of the participant’s daily activity (28–43%). The participants’ consideration of the balance exercises to be challenging also supported this result. Bearing in mind the results found in the present study, the efficacy of this training intervention seems reasonable given the positive effects on balance, gait and physical activity [28], which could be attributed to the levels and progressive nature of dynamic balance training. Establishing objective indicators of balance training not only has the benefit of improving the description of balance training but also helps establishing future dose-response relationships.

The outcomes used in the present study refer to the dynamic aspects of balance exercises, and they were chosen since dynamic control (e.g. walking) represents an essential domain within balance control [44] and balance training [14, 15]. Furthermore, the volume and intensity of activity are significant indicators within physical activity research [12, 13] and they reflect the framework of this training program (i.e. aiming to increase the amount of dynamic exercises) [30, 31]. However, an important limitation of using wearable sensors to monitor training activity is that this method only provides an absolute output of gait-related balance exercises without capturing the relative intensity (i.e. the capacity of the individual to meet the challenges of a certain exercise). Accordingly, what might be perceived as a difficult exercise for one person might not be perceived as difficult for another person with a different type or level of disability. In our study, there was a large variation in the absolute training activity (Fig. 1a, b, c) and it is likely that similar variation also occurred for the relative intensity. Therefore, it had been useful to also include data of the participants’ perception of the exercise to address the relation between absolute and relative intensity throughout the program. Furthermore, the present approach of using wearable sensors do not provide information about quality of movements (e.g. the walking pattern, instability and near fall events) and direct measures of intensity (e.g. heart rate). Therefore, the utility of wearable sensors requires further validation, and future studies including physiological indicators, objective data of movement characteristics [45, 46] and psychometric evaluations of the performance of exercises (e.g. fall risk and movement quality) [4, 5] are recommended.

An expected, nevertheless interesting finding was that training activity only increased after a period of habituation to cognitive-demanding DT exercises (i.e. between Blocks B and C, see Fig. 1a, b). Indeed, while DT training may be important due to its similarities shared with daily living, [9, 25] it is known for degrading motor performance, e.g. leading to shorter step length and decreased velocity during walking [47]. For individuals with PD, this may be specifically manifested as increased bradykinesia, particularly during the initial stages of DT exposure [9, 25]. Therefore, the pattern of training progression observed here corresponds to previous research where individuals with PD, who in general need longer time to achieve motor learning, [48] improved DT abilities after 4-6 weeks of training [49, 50]. Hence, these findings indicate that wearable sensors may adequately identify progression of motor performance of gait-related balance exercises including DT in individuals with PD.

This study has several limitations. Most importantly, our results are based on a small sample size and a specific training intervention, which may limit the generalisability of these findings. However, generalisability was not our main focus; instead, this study aimed to evaluate training activity among this particular sample across multiple measurement occasions. Furthermore, as wearable sensors only provide information about the absolute volume and intensity of gait-related balance exercises the present methodology cannot be applied to training programs focusing on stationary exercises (e.g. weight shift exercises). Although we used accelerometer cut-points developed for individuals with mild to moderate PD [41] this might not correspond to the relative intensity the individual perceive while performing the exercises. Despite these shortcomings, we believe that wearable sensors could be applied to delineating the dose-response relationship of other programs including gait-related balance exercises (e.g. home-based exercises) and be used among diverse populations with balance impairments.

## Conclusion

These findings support the feasibility of applying wearable sensors in clinical settings to gain objective informative measures of gait-related balance exercises in individuals with PD. Objective measurements of gait-related balance exercises could improve the description of training as well as help establishing future dose-response relationships. Still, this activity monitoring approach needs to be validated further in other populations and balance training programs.

## Abbreviations

DT: dual-tasking
PD: Parkinson’s disease
UPDRS: Unified parkinson’s disease rating scale
